# Variability of serum novel serum peptide biomarkers correlates with the disease states of multiple myeloma

**DOI:** 10.1186/s12014-019-9238-0

**Published:** 2019-04-23

**Authors:** Ju Bai, Yun Yang, Jianli Wang, Lei Zhang, Fangxia Wang, Aili He

**Affiliations:** 1 Department of Hematology, Second Affiliated Hospital, Medical School of Xi’an Jiaotong University, 157 Xiwu Road, Xincheng District, Xi’an, 710004 Shaanxi Province China; 2 Department of Clinical Lab, Second Affiliated Hospital, Xi’an Jiaotong University, 157 Xiwu Road, Xi’an, 710004 Shaanxi China

**Keywords:** Biomarkers, Proteomic profiling, Bone marrow microenvironment, Treatment response, Multiple myeloma

## Abstract

**Background:**

The bone marrow microenvironment provides an optimal substrate for multiple myeloma (MM) initiation and progression. The soluble component of MM niche is dynamic with the disease states of MM. We formerly employed proteomic profiling to construct a MM model. Four peptides constituting the model were selected by supervised neural network algorithm (SNN).

**Methods:**

62 Newly diagnosed (ND) MM and 64 healthy controls (HCs) were picked up for validating the distinguishing capability of the SNN model. Nano-liquid chromatography-electrospray ionization-tandem mass spectrometry was used for peptide identification. MM in different disease states and HCs were choosed for peptides relative intensities comparison. Western blot and ELISA were employed to validate the variability.

**Results:**

The sensitivity and specificity of the independent testing data set for blind validation were 93.55% and 92.19%. The relative intensities of three out of the four peptides were increased in ND and refractory and relapse patients but decreased to that level of HCs in complete remission and very good partial remission patients. Relative intensity of the remaining peptide was negatively associated with MM remission. The peptides sequencing results showed that they were derived from dihydropyrimidinase-like 2, fibrinogen alpha chain, platelet factor 4 and alpha-fetoprotein.

**Conclusions:**

The potential value of the four peptides in monitoring MM treatment response was arised from their close correlation with MM disease states.

## Background

Multiple myeloma (MM) is a malignant B cell clonal disease with a worldwide incidence of 6–7 cases per 100,000 persons per year [1]. More disturbingly, it has been noted that for some unknown reason MM displays a trend of increasing incidence [2]. Despite the great efforts in the past decades, treatment of MM is not effective and the prognosis of MM is poor. Therefore, MM is still considered largely as an incurable disease.

Cytogenetic abnormalities play a crucial role in MM pathogenesis. The bone marrow microenvironment provides an optimal substrate for MM initiation and progression [3]. MM cells and bone marrow derived stroma cells (BMSCs) profoundly influence each other’s behavior via a variety of molecular mechanisms. Among these, changes in the secretion of cytokines, growth factors, and chemokines play a prominent role in supporting cancer cell growth, survival, trafficking, and resistance to therapies [4]. Plasma cells can secrete cytokines (e.g. tumor necrosis factor alpha, transforming growth factor beta, vascular endothelial growth factor (VEGF), fibroblast growth factor-2, etc.) to allow for infiltration, growth, proliferation, adhesion, and migration of MM cells [5–9]. Moreover, binding of plasma cells to BMSCs triggers transcription and secretion of cytokines by the latter, such as interleukin-6 (IL-6), insulin-like growth factor-1 (IGF-1), VEGF and CXCL12/stromal cell derived factor-1 (SDF-1) [10–13] that mediate plasma cell growth, survival, and drug resistance, as well as bone marrow (BM) angiogenesis. However, a definite pathogenic role for the BM niche in MM remains under investigation.

The soluble component of MM niche is dynamic with the disease states of MM. Considering that the heterogeneous nature of MM and the involvement of multiple proteins in BM milieu, a combinatory approach using a panel of biomarkers is more reliable and sensitive than relying on a single protein. Mass spectrometry based proteomic profiling holds great promise for the identification of circulating biomarkers useful for a better understanding of bone marrow microenvironment of MM. Previously, we applied a comparative peptidomics method combining weak cation exchange beads (MB-WCX) and matrix assisted laser desorption ionization time of flight mass spectrometry (MALDI-TOF-MS) to analyze serum peptide profiles of newly diagnosed (ND) MM and healthy controls. A diagnostic model including four peptides (2660.65, 2900.4, 3315.96, 7763.24 Da) was constructed based on supervised neural network algorithm (SNN), which could discriminate newly diagnosed (ND) MM and healthy controls. Appreciable levels of sensitivity and specificity, 87.5% and 86.36%, respectively, were achieved in a small patient samples [14]. Therefore, to better understand the soluble component of MM niche, we used mass spectrometry based proteomic profiling to characterize the variability of serum proteome in different MM treatment durations.

## Materials and methods

### Study population

This study was approved by Ethics Committee of the Second Affiliated Hospital of Xi’an Jiaotong University. 62 ND patients, 38 complete remission and very good partial remission (CR&VGPR) patients and 43 refractory and relapse (RR) patients hospitalized in our hospital were recruited for the study (2010.1–2016.12) (Characteristics details were showed in Table 1). Their diagnoses were made according to the International Myeloma Working Group (IMWG) criteria [15]. Treatment response was assessed based on IMWG uniform response criteria for MM [16]. All MM cases were treated with VAD (Vinorelbine, Pirarubicin and Dexamethasone), CHOP (Cyclophosphamide, Vinorelbine, Pirarubicin and Dexamethasone) regimen and oral thalidomide. 64 HCs (age range 45–70, median age 57.5 years old, male/female 34/30) were enrolled. They had no finding of abnormal symptoms or test results in physical examination. All patients and healthy controls signed the informed consent form.

**Table 1:** Clinical features of patients in different MM groups before chemotherapy

Clinical features	ND cases (n = 62)	CR&VGPR cases (n = 38)	RR cases (n = 43)
Sex			
Male	34	21	27
Female	28	17	16
Age (year)	56.5 (44–73)	54.5 (44–71)	57.5 (45–73)
WBC (× 10^9^/L)	5.7 (2.7–14)	5.3 (3.2–11.5)	6.2 (2.3–15.3)
Hb (g/L)	83 (52–101)	82 (63–132)	72 (48–95)
PLT (× 10^9^/L)	190 (23–356)	202 (89–387)	183 (56–361)
Subtype			
IgG type	32	20	26
IgA type	12	8	8
IgM type	0	0	0
Light chain type	8	5	5
Non-secretory type	10	5	4
MM cell > 10%	62	38	43
ESR↑	53	31	36
Glb (> 30 g/L)	52	33	39
Renal failure (serum creatinine ≥ 2 mg/100 mL)	13	6	9
Hypercalcemia	15	6	8
Bence-Jone protein	21	5	9
LDH↑	35	19	28
Bone lesions (lytic lesions, pathologic fractures or severe osteopenia)	50	31	35

This table showed clinical features of MM patients in different groups at the time of diagnosis

*ESR* erythrocyte sedimentation rate, *LDH* lactate dehydrogenase

### Serum peptides enrichment and MALDI-TOF data acquisition

The procedure of serum peptides enrichment and MALDI-TOF data acquisition were described previously [14]. The serum peptides were fractionated using weak cation exchange magnetic-beads (MB-WCX) (ClinProt purification reagent sets; Bruker Daltonics, Bremen, Germany) with a magnetic separator. Mixture with the eluted sample and matrix was spot onto a MALDI-TOF mass spectrometry target (AnchorChip™, Bruker Daltonics) for peptide profiling acquisition in Microflex mass spectrometer (Bruker Daltonics). Cilnplot standard was used for mass calibration. The scan range was 0.7–10 KD. Peptide profiling by different mass to charge ratio was obtained through FlexControl2.2 software. The coefficient of variability less than 30% indicated that the system was stable and reliable.

### Peptides identification

A nano-liquid chromatography-electrospray ionization-tandem mass spectrometry (nano-LC/ESI-MS/MS) system was used for peptide sequencing and peptide identification. The detailed procedure was done according to previous description [17]. The peptide solutions purified by magnetic beads, were loaded to a C18 trap column (nanoACQUITY) [180 μm × 20 mm × 5 μm (symmetry)]. The flow rate was 15 μL/min. Then the desalted peptides were analyzed by C18 analytical column (nanoACQUITY) [75 μm × 150 mm × 3.5 μm (symmetry)] at a flow rate of 400 nl/min for 60 min. The mobile phases A (5% acetonitrile, 0.1% formic acid) and B (95% acetonitrile, 0.1% formic acid) were used for analytical columns. The gradient elution profile was as follows: 5%B–45%B–80%B–80%B–5%B–5%B in 60 min. The spray voltage was 1.8 kV. MS scan time was 60 min. Experimental mode were data dependent and dynamic exclusion, scilicet MS/MS spectra were limited to two consecutive scans per precursor ion within 10 s followed by 90 s of dynamic exclusion. Mass scan range was from m/z 400 to 2000. MS scan used Obitrap, resolution was set at 100,000. CID and MS/MS scan employed LTQ. In MS spectrogram, we selected single isotope composed of 10 ions with strongest intensity as parent ion for MS/MS. Single charge was excluded and not as parent ion. We applied data analysis software Bioworks Browser 3.3.1 SP1 for Sequest™ retrieving. Retrieval Database was International Protein Index (IPI human v3.45 fasta with 71,983 entries). Parent ion error and fragment ions error were set at 100 ppm and 1 Da, respectively. Digested mode was non-digested and variable modification was methionine oxidation. Positive protein identification was accepted for a peptide with Xcorr of greater than or equal to 3.50 for triply charged ions and 2.5 for doubly charged ions, and all with ΔCn ≥ 0.1, peptide probability ≤ 1e−3.

### Western blot analysis

Immunoblotting was performed as described previously [17]. Cell pellets lysed in lysis buffer (10 mM Tris-HCl (pH 7.4), 5 mM MgCl2, 1% Triton X-100, 100 mM NaCl, 10 mM NaF, 1 mM Na_3_VO_4_) and protease inhibitor cocktail. Then cellular proteins were subjected to sonication. The proteins were separated sodium dodecyl sulfate polyacrylamide gel electrophoresis (SDS-PAGE) and transferred to polyvinylidene fluoride membranes (Roche Diagnostics Corporation, Indianapolis, Indiana United States), which were incubated with the appropriate primary antibodies. Immunoreactivity was detected by horseradish peroxidase-labeled secondary anti-bodies. Color development was done by chemiluminescence substrates (7seapharmtech, Shanghai, China). Image Reader Tano-5500 (Tano, Shanghai, China) was used to document the results. 2D Densitometry Image Analyzer IPP 7.0 software (Tano, Shanghai, China) was applied to quantify the images. Samples were measured in triplicate and then mean densitometry was calculated.

### Enzyme linked immunosorbent assay

Enzyme linked immunosorbent assay (ELISA) was used to detect the level of fibrinogen alpha chain, platelet factor 4 (PF4) and vascular endothelial growth factor (VEGF) in the serum of 62 ND MM patients and 64 HC. The experiments were conducted following the manufacturers’ instructions of ELISA kit (R&D, USA).

### Statistics

Flexanalysis 3.0 software was used to normalize original mass spectrum, including smoothing and substrate baseline. Acquisition of differentially expressed peptides and statistical analysis were obtained by statistical algorithm built-in Clinprotools 2.2 software. The differences of peptides relative intensities in different groups were analyzed through ANOVA test. The statistical software SPSS 17.0 was used to do independent sample *t* test comparative analysis. We performed correlation analysis by linear regression model. The statistical significance (*p* < 0.05) was determined by the assumption of equally deviation and two-sided distribution. Significant level was adjusted to 0.0083(0.05/4(4 − 1)/2) when multiple comparisons were made among the four groups.

## Results

### Blind validation of MM SNN model

**Fig. 1:**
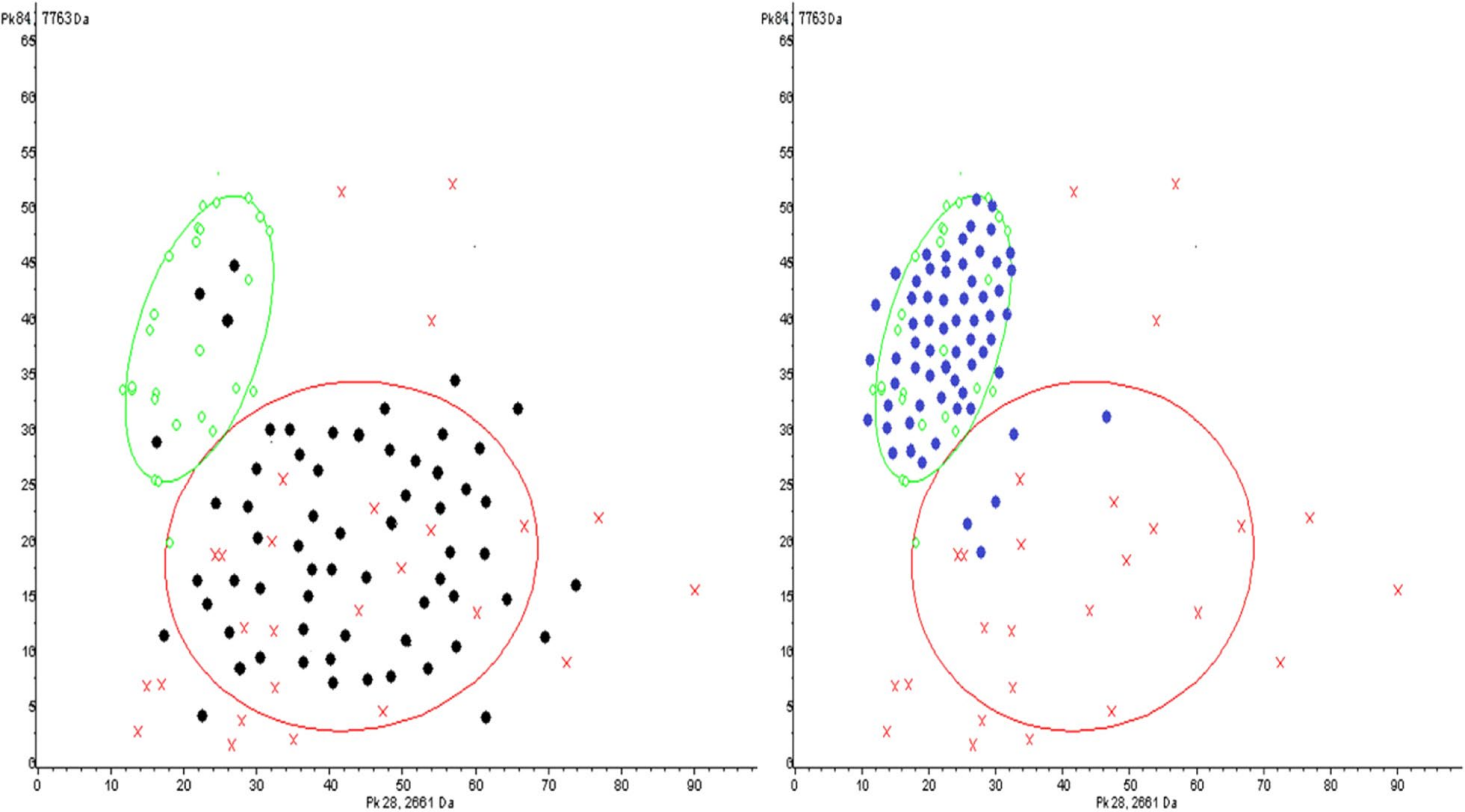
Serum specimens from 62 ND MM and 64 HCs were used for SNN model blind validation. 58 cases of MM were correctly assigned to MM group (93.55% sensitivity). 59 HCs were correctly identified as the non-diseased, 5 cases had been wrongly judged as MM (92.19% specificity). Green circle: HCs; Red cross: MM ND patients; Black dot: MM ND patients in validation group; Blue dot: HCs in validation group

62 newly diagnosed MM and 64 HCs serum specimens were used for blind validation of MM SNN model which was established by our group in previous study. 58 MM were correctly identified as the diseased, so sensitivity of SNN model was 93.55% (Fig. 1a). 59 HCs were correctly assigned to the non-diseased group, 5 cases was misdiagnosed as MM patients, indicating the specificity was 92.19% (Fig. 1b).

### Comparison of the four peptides relative intensities

The multiple comparisons of relative intensities of the four peptides were made in MM with different diease states (ND, CR&VGPR and RR) and HC. The results were summarized in Table 2. The relative intensities of three peptides (2660.65, 2900.4 and 3315.96 Da) were increased in ND and RR patients but decreased to that level of HCs in CR&VGPR patients (*p* = 0.0000505; *p* < 0.000001; *p* < 0.000001). The relative intensity of the rest of peptide (7763.24 Da) was negatively associated with MM remission (*p* < 0.000001).

**Table 2:** Relative intensity of the four peptides in MM different groups and healthy control

Peaks (Da)	ND group (n = 62)^a^	HC group (n = 64)	CR&VGPR group (n = 38)	RR group (n = 43)
2660.65	47.73 ± 28.41	24.58 ± 11.3	26.73 ± 12.43	46.37 ± 27.54
2900.4	4.96 ± 2.12	1.31 ± 0.35	1.44 ± 0.38	5.08 ± 2.58
3315.96	7.48 ± 5.08	2.08 ± 1.16	1.94 ± 0.65	8.55 ± 5.78
7763.24	2.09 ± 1.69	8.86 ± 3.25	7.63 ± 2.01	1.91 ± 1.25

^a^“n” stands for the number of patients

### Identification of peptides

**Fig. 2:**
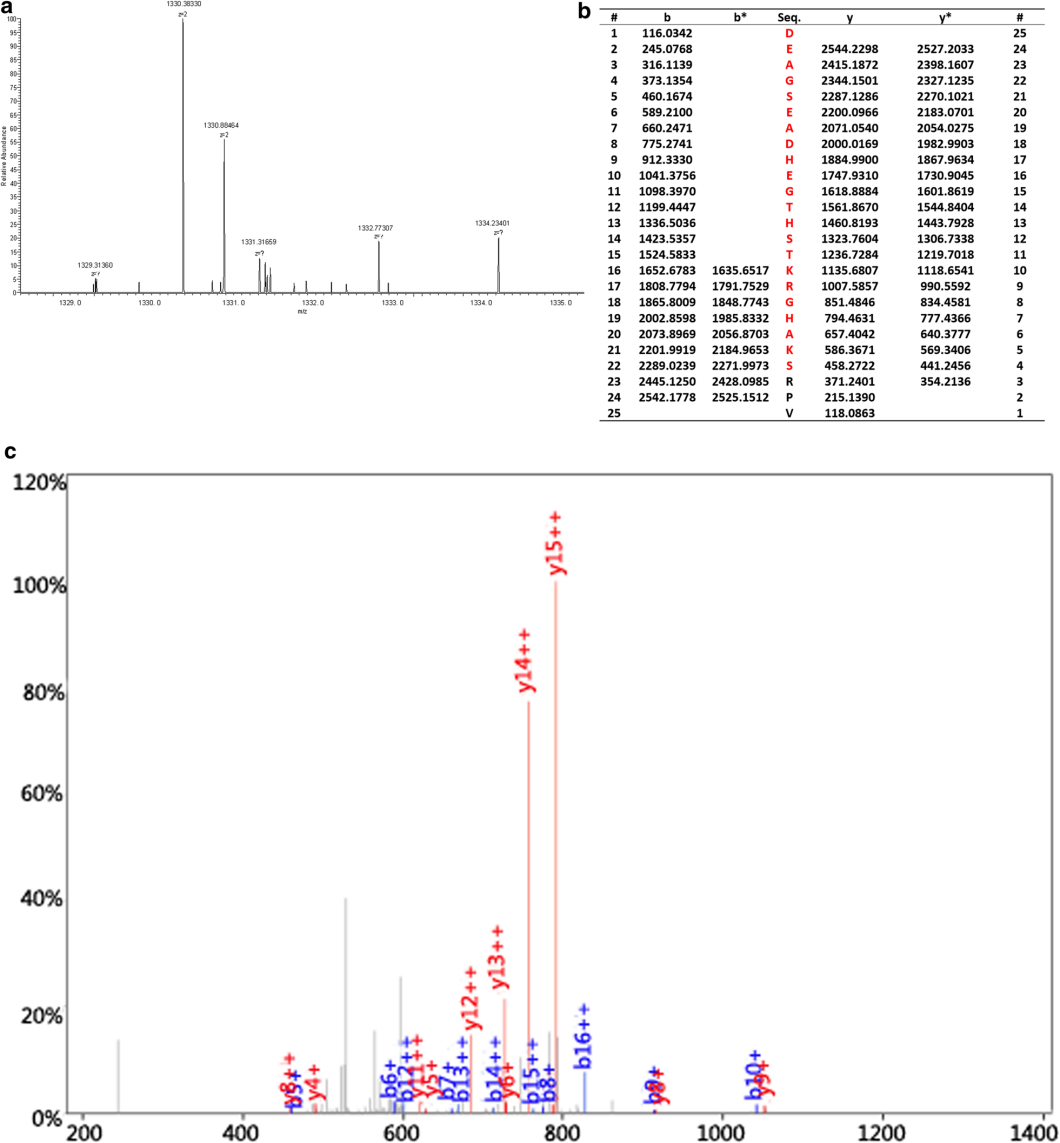
MS/MS map of peptide with MW of 2660.65 Da. **a** The enlarged picture of peptide with MW of 2660.65 Da. **b** The b and y ions spectra used to identify the peptide with MW of 2660.65 Da. **c** The sequence of the peptide with MW of 2660.65 Da

**Fig. 3:**
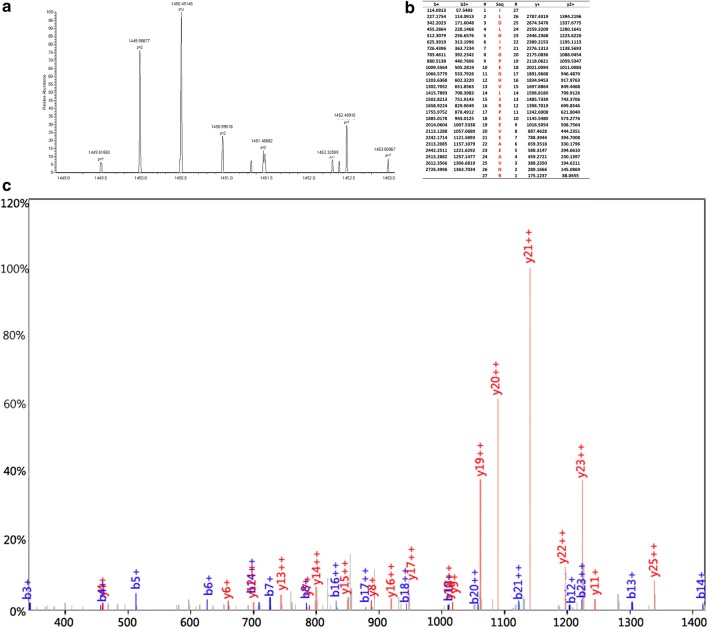
MS/MS map of peptide with MW of 2990.4 Da. **a** The enlarged picture of peptide with MW of 2990.4 Da. **b** The b and y ions spectra used to identify the peptide with MW of 2990.4 Da. **c** The sequence of the peptide with MW of 2990.4 Da

**Fig. 4:**
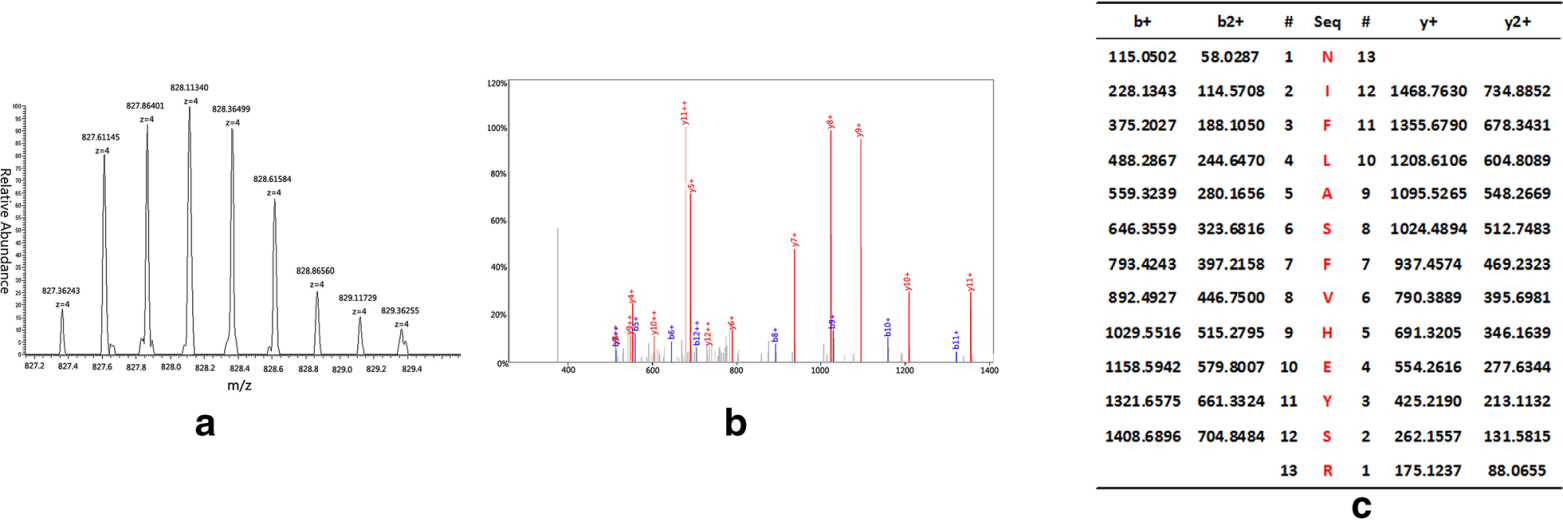
MS/MS map of peptide with MW of 3315.96 Da. **a** The enlarged picture of peptide with MW of 3315.96 Da. **b** The b and y ions spectra used to identify the peptide with MW of 3315.96 Da. **c** The sequence of the peptide with MW of 3315.96 Da

**Fig. 5:**
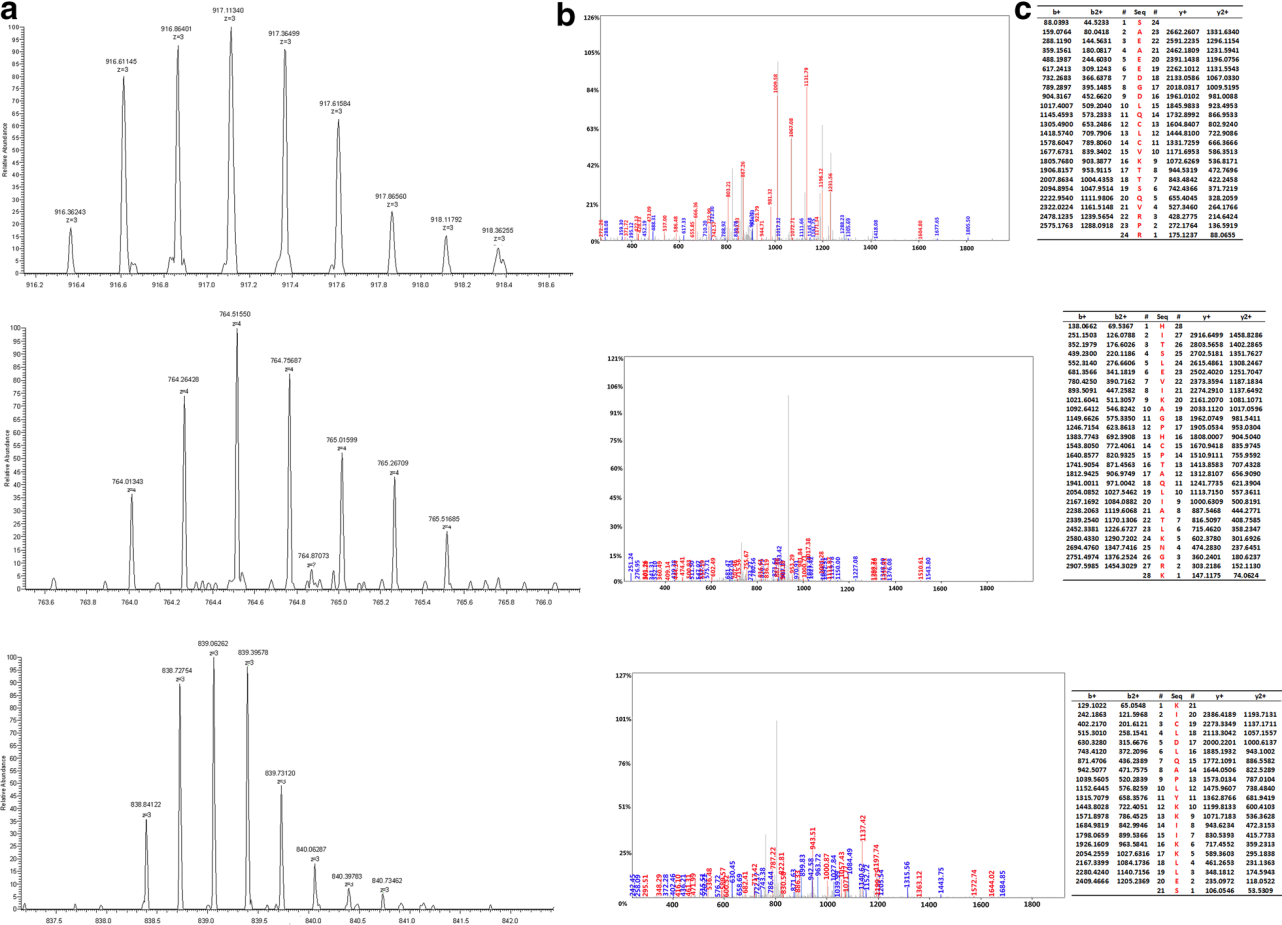
MS/MS map of peptide with MW of 7763.24 Da. **a** The enlarged picture of peptide with MW of 7763.24 Da. **b** The b and y ions spectra used to identify the peptide with MW of7763.24 Da. **c** The sequence of the peptide with MW of 7763.24 Da

We adopted the HPLC-MS/MS to identify the four peptides in the SNN diagnostic model. The four peptides from the diagnostic pattern for MM were derived from dihydropyrimidinase-like 2, fibrinogen alpha chain, platelet factor 4 and alpha-fetoprotein, respectively (Table 3, Figs. 2, 3, 4, 5).

**Table 3:** Peptides sequencing and identification results

Molecular weight (Da)	Peptide name	International Protein Index	Amino acid sequence
2660.65	α-Fibrinogen	IPI00021885	DEAGSEADHEGTHSTKRGHAKSRPV
2990.4	Dihydropyrimidinase-like 2	IPI00106642.4	ILDLGITGPEGHVLSRPEEVEAEAVNR
3315.96	Alpha-fetoprotein	IPI00022443	FLGDRDFNQFSSGEKNIFLASFVHEYSR
7763.24	Platelet factor 4	IPI00022446	EAEEDGDLQCLCVKTTSQVRPRHITSLEVIKAGPHCPTAQLIATLKNGRKICLDLQAPLYKKIIKKLLES

### Validation of protein fragments by western blot

**Fig. 6:**
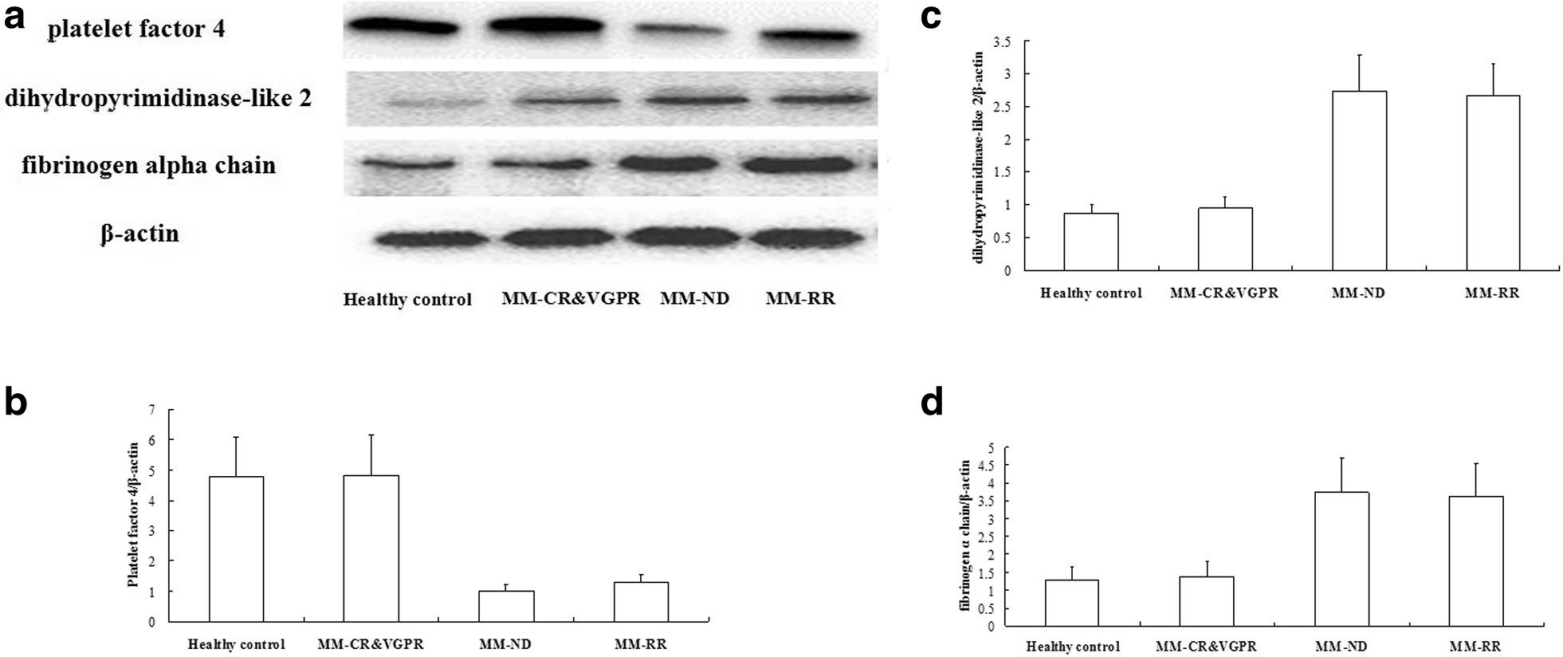
Validation of protein fragment by Western blot analyses. **a** Expression levels of the fibrinogen alpha chain and dihydropyrimidinase-like 2 differ among the four groups of HC, MM-ND, and MM-RR. PF4 immunoreactive bands show that weak or no band was detected in ND and RR MM cases. **b** Densitometry comparison of PF4 protein relative to β-actin as determined by Western blot analyses in (**a**). **c** Densitometry comparison of dihydropyrimidinase-like 2 protein relative to β-actin as determined by Western blot analyses in (**a**). **d** Densitometry comparison of fibrinogen alpha chain protein relative to β-actin as determined by Western blot analyses in (**a**)

Fibrinogen alpha chain and dihydropyrimidinase-like 2 immunoreactive bands showed that protein expression in MM with different disease states and HC samples were quite heterogeneous (*p* = 0.0034, *p* = 0.0067, Fig. 6a, c, d). It is noticeable that PF4 protein levels in ND and RR MM cases were higher than those in CR&VGPR and HC (*p* = 0.0031, Fig. 6a, b).

### Determination of serum PF4, fibrinogen alpha chain and VEGF

ELISA was used to determine the serum PF4, fibrinogen alpha chain and VEGF content in 62 ND MM patients and 64 HC. The PF4 concentration was significantly increased in HC (1.43 ± 0.77 μg/L) when comparing with ND MM (0.67 ± 0.18 μg/L) (*p* = 1.86 E−6). Higher concentrations of fibrinogen alpha chain and VEGF were seen in MM than in HC samples (276.78 ± 194.75 μg/L vs. 30.44 ± 22.15 μg/L, *p* = 1.82E−12; 669.69 ± 137.81 ng/L vs. 120.75 ± 36.96 ng/L, *p* = 7.28E−29).

**Fig. 7:**
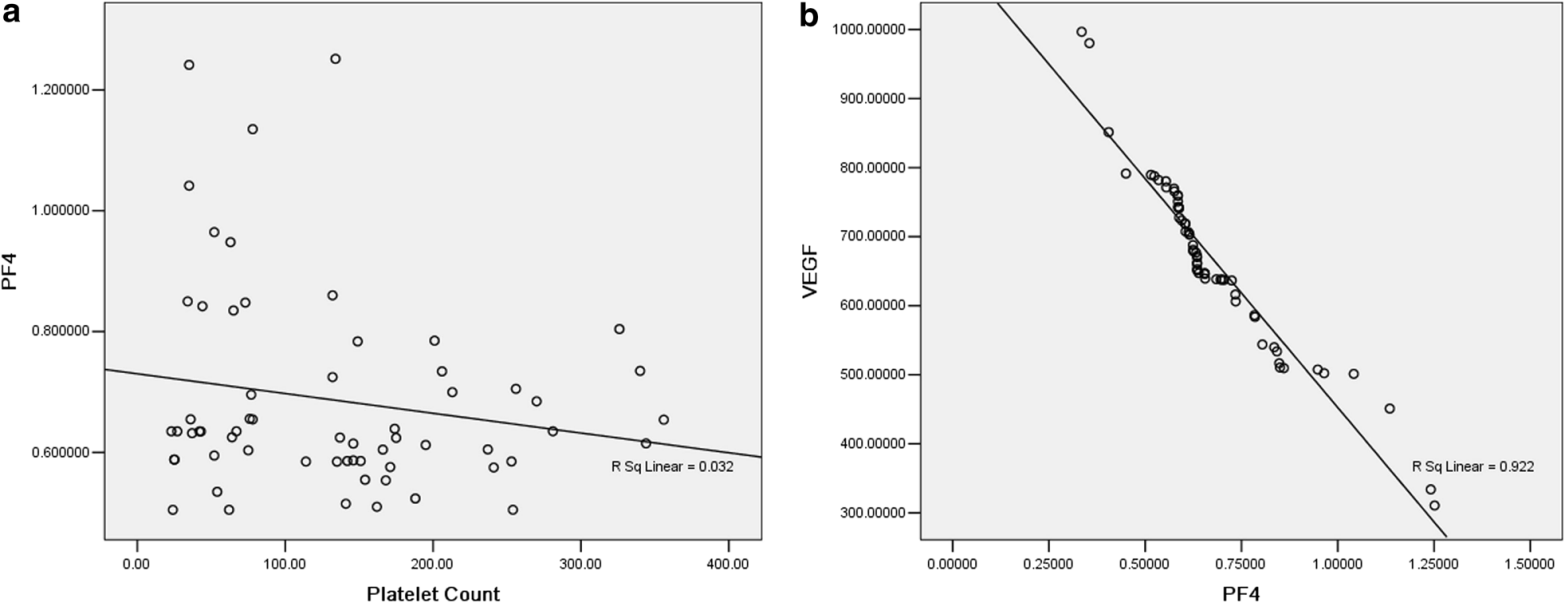
Correlation analysis between PF4 and platelet count as well as VEGF. **a** Correlation analysis between serum PF4 and platelet count in ND MM (r = 0.179; *p* = 0.165). **b** Correlation analysis between serum PF4 and serum VEGF in ND MM (r = − 0.960; *p* = 1.108e−014)

We classified 62 ND MM patients into thrombocytopenia group and platelet normal group in order to exclude the impact of platelet count upon PF4. PF4 concentrations were similar in the two groups (0.65 ± 0.18 μg/L vs. 0.69 ± 0.17 μg/L, *p* = 0.55). Further correlation analysis of platelet count and serum PF4 level revealed the correlation coefficient was 0.18 (*p* = 0.17) (Fig. 7a), indicating no relationship was found between them. However, serum PF4 and serum VEGF appear in an obvious negative correlation. The correlation coefficient was − 0.96 (*p* = 1.11E−14) (Fig. 7b).

## Discussion

The exchange of MM cells with the bone marrow microenvironment also plays a pivotal role in MM pathogenesis. Since the malignant B-cells produce large amount of proteins and these proteins are readily detectable in circulation, mass spectrometry-based proteomic profiling, with its high throughput and sensitivity, holds great promise to identify biomarkers useful for the molecular mechanisms of MM. Such biomarkers may also serve as therapeutic target or indicator for disease state of MM patients. In the present study, an independent testing data set composed of 62 newly diagnosed MM and 64 healthy control serum specimens was used for blind validation of previously established MM SNN model [14]. The sensitivity and specificity reached the encouraging levels of 93.55% and 92.19%, respectively, suggesting that SNN model has a decent power in distinguishing MM patients from healthy individuals. Further efforts for improvement and standardization of the procedure are required for potential clinical application of this SNN model.

Interestingly, the MM candidate biomarkers identified by us and other researchers are distinct. This discrepancy is partially attributable to the different methods used for peptides enrichment and different analytic algorithm and software in data analysis and interpretation [18–22]. The divergent results from different studies suggested that a large pool of differentially expressed proteins is present in MM samples, and the searching of these differences is far from exhausted.

As part of the validation efforts, comparisons of the peptides relative intensity differences were further made in MM with different response states and the healthy control cases. Three peptides with higher relative intensities in ND and RR cases were decreased to that level of HCs in CR&VGPR patients. Relative intensity of the remaining peptide was negatively associated with MM remission. All the aforementioned peptides decreased significantly after effective treatment. This is probably the result of the direct cytotoxic effect of the drugs on plasma cells, leading to a reduction in the release of cytokines from the myeloma cells as well as a reduction in their proliferation. Our work shows that, apart from well-identified MM cell-derived soluble factors, the dynamic changes in these peptides along with the progression or remission of the disease raised a possibility for their application to monitoring of disease state and assessment of therapeutic effects.

Fibrinogen alpha chain (FGA), together with beta and gamma chains, is circulating fibrinogens. Numerous studies have shown that many malignancies own over expression of fibrinogen, and high fibrinogen is correlated with disease-free, distant disease-free and overall survival [23–28]. In current study, we found an inverse correlation between the relative intensity of FGA peptide fragment and the remission of the disease. The relative intensity raised again as MM patients relapsed. This finding was validated by Western blot analysis. Endogenous protein substrates are generated ex vivo by endogenous proteases and these degradation products can be detected by MALDI-TOF-MS called human serum peptidome. Peptides are degraded in coagulation and complement cascades by high activity of proteolytic enzymes, always occurring on serine proteases cleavageesites [29]. We speculated that the identified peptide (D605–V629) was a plasmin-generated proteolytic fragment. Therefore, it is likely that raised enzymatic activity may constantly cleave the FGA C-terminal end to form the over expression of serum peptide fragments (2660.65 Da).

Dihydropyrimidinase-like 2, namely collapsin response mediator protein-2 (CRMP-2), was implicated in the development of nervous system and highly expressed in mouse neuroblastoma Neuro2a cell lines [30]. Wu et al. analyzed the proteome of colorectal carcinoma (CRC) cell lines and found that CRMP-2 can be used as a potential marker for CRC [31]. A research shows that CRMP-2 phosphorylation and mRNA splicing was correlated with carcinoma cell migration and invasion [32]. In current study, the expression of dihydropyrimidinase-like 2 was relevant to the remission of the disease. Thus, CRMP-2 can be used for monitoring response to treatment in MM patients.

The peptide with molecular weight of 7763.24 Da is identified as PF4. ELISA measurement reveals that its levels in serum significantly decreased in ND group compared to HC group. After comparing the differences between the thrombocytopnia group and the platelet normal group in MM patients and conducting the correlation analysis between the PF4 level in serum and the platelet count, it was found that this difference was not due to alterations in platelet count. Interestingly, Cheng’s group reported that Chromosome 4q deletion in clonal plasma cells were present in 70–74% monoclonal gammopathy of undetermined significance (MGUS) and MM cases, suggesting that this region may harbor tumor suppressor gene (TSG). Further analysis found that platelet factor 4 was the candidate TSG and down-regulated in MM due to promoter hypermethylation [33]. Comparing with normal peripheral blood plasma cells, PF4 mRNA expression was not detectable in MM cell lines and a significant down regulation was observed in newly diagnosed MM. Thus, data from this and other studies strongly suggested that PF4 inactivation may be involved in MM pathogenesis, and PF4 could be a potential therapeutic target for MM.

VEGF stimulates bone marrow stromal cells to produce IL-6 that can promote the differentiation of normal B cells to plasma cell [34, 35]. VEGF binding to VEGF receptor is mediated by heparin. PF4 also has a strong heparin-binding ability. Thus PF4 can compete with VEGF in binding to heparin, by which PF4 may block the VEGF interaction with VEGFR, and hence, its angiogenic activity [36]. In our study, serum peptidome and immunoblotting results showed that PF4 expression was positively related with MM response. Furthermore, serum PF4 and VEGF levels were negatively correlated. Physical interaction between PF4 and VEGF provided a molecular mechanism for the tumor suppressive activity of PF4. Further studies along this direction may help our understanding on the tumorigenesis of MM.

## Conclusions

In summary, we showed that the MM SNN model possessed decent efficiency in the differential diagnosis of MM and healthy controls. The four peptides should be considered strong candidate biomarkers of MM. PF4 is down regulated in newly diagnosed MM patients and has great potential of tumour-suppressive function in MM development. These candidate biomarkers have shown dynamic changes along the progression or remission of MM. Their levels may be used for monitoring of disease state and assessment of therapeutic effects.

## Abbreviations

MM: multiple myeloma
SNN: supervised neural network algorithm
ND: newly diagnosed
HCs: healthy controls
BMSCs: bone marrow derived stroma cells
VEGF: vascular endothelial growth factor
IL-6: interleukin-6
IGF-1: insulin-like growth factor-1
SDF-1: stromal cell derived factor-1
CR&VGPR: complete remission and very good partial remission
RR: refractory and relapse
MB-WCX: weak cation exchange magnetic-beads
nano-LC/ESI-MS/MS: nano-liquid chromatography-electrospray ionization-tandem mass spectrometry
ELISA: enzyme linked immunosorbent assay
PF4: platelet factor 4
FGA: fibrinogen alpha chain
CRMP-2: collapsin response mediator protein-2
CRC: colorectal carcinoma
TSG: tumor suppressor gene
SDS-PAGE: sodium dodecyl sulfate polyacrylamide gel electrophoresis
BM: bone marrow
IMWG: International Myeloma Working Group
MALDI-TOF-MS: matrix-assisted laser desorption/ionization time of flight mass spectrometry
MGUS: monoclonal gammopathy of undetermined significance
